# Changes in Habitat Structure May Explain Decrease in Reintroduced Mohor Gazelle Population in the Guembeul Fauna Reserve, Senegal

**DOI:** 10.3390/ani2030347

**Published:** 2012-08-08

**Authors:** Eulalia Moreno, Abibou Sane, Jesús Benzal, Belén Ibáñez, Joaquín Sanz-Zuasti, Gerardo Espeso

**Affiliations:** 1 Department of Functional and Evolutionary Ecology, Estación Experimental de Zonas Áridas, CSIC, Carretera de Sacramento s/n, La Cañada de San Urbano, E-04120 Almería, Spain; E-Mails: jbenzal@eeza.csic.es (J.B.); belen@eeza.csic.es (B.I.); gerardo@eeza.csic.es (G.E.); 2 Direction des Parc Nationaux, PB 5135 Dakar-Fann, Senegal; E-Mail: abibousane@gmail.com; 3 Camino de Zafara, 8, Tudera-Fariza, E-49214 Zamora, Spain; E-Mail: j.sanz-zuasti@hotmail.com

**Keywords:** evaluation of reintroduction, habitat structure, *Nanger dama mhorr*, post release monitoring, Senegal

## Abstract

**Simple Summary:**

The reintroduction of plants and animals to the wild is an important technique to save endangered species from extinction. To perform post release monitoring is crucial to evaluate reintroduction outcomes. A Mohor gazelle reintroduction programme took place in Senegal in 1984. We attempt to explain why the size of the reintroduced gazelle population has diminished in recent years. We suggest that changes in habitat structure occurred over time and have very likely reduced the amount of suitable habitat for this species.

**Abstract:**

Reintroduction is a widespread method for saving populations of endangered species from extinction. In spite of recent reviews, it is difficult to reach general conclusions about its value as a conservation tool, as authors are reluctant to publish unsuccessful results. The Mohor gazelle is a North African gazelle, extinct in the wild. Eight individuals were reintroduced in Senegal in 1984. The population grew progressively, albeit slowly, during the first 20 years after release, but then declined dramatically, until the population in 2009 was estimated at no more than 13–15 individuals. This study attempts to determine the likelihood of gazelle-habitat relationships to explain why the size of the gazelle population has diminished. Our results show that the Mohor gazelle in Guembeul is found in open habitats with less developed canopy where the grass is shorter, suggesting the possibility that changes in habitat structure have taken place during the time the gazelles have been in the Reserve, reducing the amount of suitable habitat. Reintroduction design usually concentrates on short-term factors that may affect survival of the released animals and their descendants (short-term achievement), while the key factors for assessing its success may be those that affect the long-term evolution of the population.

## 1. Introduction

Reintroduction has emerged as a widespread method for saving endangered species from extinction, representing a particularly suitable conservation strategy when *in situ* and *ex situ* measures have been shown to be insufficient. However, published research on its outcome has been limited [1], as most authors prefer to publish their “successful” results than to report failures or even uncertainties. In recent years reintroduction reviews have been increasing [2,3,4,5]. Most of them strongly recommend carrying out periodically post-release monitoring as well as reporting of results, as differences over time in the known and unknown outcomes of reintroduction programs (success, failure, and uncertain) have been shown. Reviews also show that reintroduction programs do not have high success rates (26% of success reported by Fischer & Lindenmayer [1]; 42% of success reported by Germano & Bishop [4]; 46% of success reported by Sheean and co-workers [5]), particularly when captive-bred animals are used. Captive breeding seems to induce significant evolutionary changes in ways that compromise fitness in natural environments [6,7]. 

The Mohor gazelle (*Nanger dama mhorr* Bennett, 1833) is the largest gazelle species known (males: 40–75 kg; females: 35–40 kg [8]). It is a North African species believed to be extinct in the wild [8]. Its range coincided more or less with the oceanic and suboceanic Atlantic Sahara, a cold-current coastal attenuated desert comprising a sub-littoral zone where steppes and acacia woodlands abound. The species inhabited areas with sparse vegetation [8], grazing gramineous or non-graminid herbaceous plants, and browsing the foliage of ligneous species, which played a particularly important role in the ecological requirements of this species [9]. Two more subspecies are widely recognized within *N. dama: N. d. dama* (in the western and central Sahel) and *N. d. ruficollis* (in the eastern Sahel; see [8] for a review). In Senegal, the Mohor gazelle has been known since at least the 18th century in the Sahelian zone [10]. Sournia & Dupuy [11], however, thought that it was only a dry season visitor (see also [12]). An *ex situ* conservation programme began at “La Hoya” Experimental Field Station (EEZA-CSIC) in Almería (Spain) in 1971, and its world’s captive population is currently about 180 individuals [13]. This species was first reintroduced in Senegal in 1984 [14]. Eight individuals (2 males: 6 females) were taken from Almería to the Réserve Spéciale de Faune de Guembeul (RF Guembeul) in Senegal. The population grew gradually to around 49 individuals in 2002 [15]. But due to unknown causes, the population has declined dramatically in last ten years, the species having disappeared from the western part of the Reserve according to information provided by rangers. In 2009, the population size was 15 individuals [16], some authors having recommended a reinforcement of the species [8]. Along with the Mohor gazelles, a small group (1 male: 2 females) of dorcas gazelles were moved from La Hoya Experimental Field Station to RF Guembeul in 1984 as well, but all of them died from traumatic accidents during the first two days after arrival [17].

Another reintroduction an attempt took place in Senegal in 1999, when eight (3 males: 5 females) Scimitar-horned Oryx (*Oryx dammah*) from the Hai Bar Zoo Reserve in Israel were transferred to RF Guembeul [18]. In 2002, two males and two females were added to this founder population from the Vincennes Zoo in Paris [19]. Contrary to the Mohor gazelles, the Scimitar-horned oryx population at the Reserve appears to be growing, albeit slowly, with normal births each year [20], and was comprised of 40 individuals in 2009 [16]. Comparison of the outcomes of the two reintroduction initiatives made the need to investigate the likely causes of the decline in the Mohor population evident. In 2010, the “Organismo Autónomo Parques Nacionales” (a dependency of the Spanish Ministry of Agriculture and Environment) and UNESCO funded a project to investigate the likely reasons for the decrease in the Mohor gazelle population observed in RF Guembeul, in agreement with the Senegalese “Direction des Parcs Nationaux” (DPN). 

Many factors have been shown to have an effect on the success/failure of reintroduction projects [7,21,22,23], such as availability of suitable habitat to cover the vital requirements of animals [24,25,26,27]. In this paper we examined gazelle-habitat relationships in the Réserve Spéciale de Faune de Guembeul. As the species used to live in Sahelian grasslands, savanna and sub-desert steppes, where the presence and density of trees appears to condition its distribution [28], we presently test the hypothesis that Mohor gazelle habitat preferences could partially explain its distribution (presence *vs.* absence) in Guembeul, a hypothesis which would provide a plausible explanation for the current decline of the species observed in this Reserve. We suggest that it is very likely paralleled by changes in habitat structure at RF Guembeul in the last 9–10 years. If this hypothesis is true, we would expect Mohor gazelles to be absent from the most vegetated, covered areas in Guembeul, the species preferably inhabiting its simplest structured habitat. 

## 2. Methods

**Figure 1:**
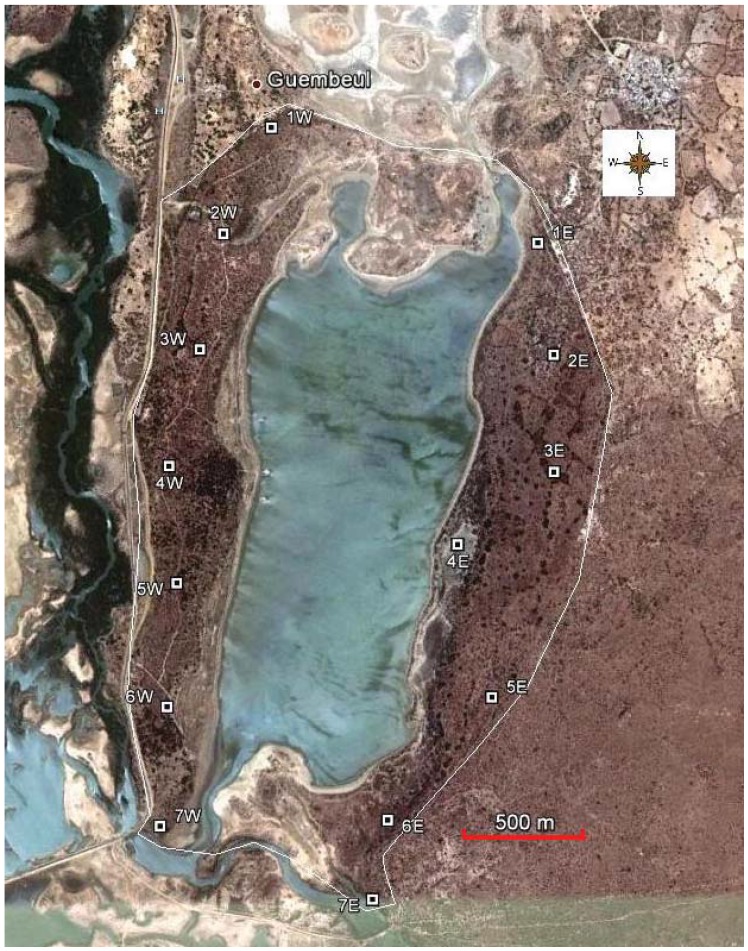
Map of the Reserve of Fauna of Guembeul showing plots where habitat structure has been sampled (15°55'N; 16°28'W). The central lagoon divides the Reserve into a western part and an eastern part, each of them containing seven sampling plots (1W–7W; 1E–7E). The white line around denotes a perimetral fence. Photo taken from Google©.

The RF Guembeul (a 720-ha fenced off area; 15°55'N; 16°28'W) is about 12 km south of Saint Louis (Sahelian zone in northern Senegal). Created in 1983, it is one of the three connected reserves in the network of lagoons and creeks which follow the Senegal River to its mouth. It was set aside primarily to protect a variety of resident and migrant water birds. In 1986, it was designated as a Ramsar Wetlands. The centerpiece of the Reserve (about 1/3 of its total area) is a large lagoon which divides RF Guembeul in two (called “West” and “East” for the purposes of this study, Figure 1). It is in a shallow depression with sandy shores surrounded by thorn-bush savannah dominated by *Acacia* sp. and *Balanites aegyptiaca* [29], and scattered *Boscia senegalensis, Salvadora persica* and *Commiphora africana* shrubs, among others. *Opuntia tuna* thickly covers some parts of the Reserve. In the eighties, it was restricted to a small area in the southwest [14], but as an exotic weed, this cactus species has become more widespread, especially in the West where it appears quite evenly distributed.

Reintroduced Mohor gazelles were part of the species EEP (European Endangered Species Programme), and were identified with the following studbook numbers [13]: ND176, ND229 (males), ND39, ND114, ND172, ND191, ND241, ND247 (females). Female ND247 died during transport. Upon arrival, a breeding group was placed in a 400-m^2^ pen (male ND176 and all of the females), and the remaining male (ND229) was kept alone in a 100-m^2^ pen. Details of the evolution of the stock released until 1992 can be found in [14].

We made a thorough review of the literature in an attempt to find out as much information as possible on how the released stock has evolved since its reintroduction in 1984. However, published information is scattered and sometimes contradictory, with gaps for which there is no information at all. Published information on the evolution of the Scimitar-horned oryx reintroduced in RF Guembeul was also reviewed for purposes of comparison.

Fieldwork was undertaken in 2011. To study habitat structure, 14 plots were selected (Figure 1). Seven were located in the West of the Reserve and seven in the East, where the remaining population of gazelles has been observed by rangers in recent years. Plots were sampled twice (once in January and once in November). Every plot consisted of a square 25 m per side separated from the closest plot by about 500 m. Each plot was geo-referenced (information available upon request). The following predictor variables were taken for each plot: tree cover (percentage), grass cover (percentage), *Opuntia tuna* cover (percentage), bare ground cover (percentage), mean tree height, mean grass height, minimum browsing height and species richness (defined as the number of different species of trees and ligneous bushes inside the plot). Data on gazelles were collected in two censuses on different days (16 and 17 November) beginning at 06:00 AM by means of a “census party” of nine people moving together from south to north over East RF Guembeul. They were distributed horizontally from the shore of the lagoon to the outer (perimetral) fence (about 600 m wide). Each person was separated from his neighbour by a distance such that each could see the one on both right and left. We were therefore sure that all animals were seen by at least one person in the census party. We recorded the number of individuals seen (Mohor gazelle or Scimitar-horned oryx), the GPS coordinate where it was observed, and whether they were solitary or in a group.

The Mohor gazelle is considered to be absent from the West of Guembeul, as none have been observed by RF Guembeul rangers in the last few years. Counts of Scimitar-horned oryx were made on 16–17 November in one-day drives and walking (another day), always at dusk. As East and West Guembeul were completely separated by the central lagoon in November, we were sure that individuals counted early in the morning in the East were different from those counted at dusk in the West, because they are unable to cross the water. In accord with previous rangers’ information, we found no Mohor gazelle in West RF Guembeul.

We performed a General Discriminant Analysis to test for differences in habitat structure between East and West RF Guembeul. To control the probability of a sampling date effect (January *vs.* November), we included this variable as a categorical predictor. To check for lack of relationships between sampling date and significant continuous predictors (plot variables), we tested their interactions. For reaching normalization, variables taken as percentages were arcsine transformed and linear variables were log-transformed. Analysis was performed using Statistica 7.0 [30]. 

## 3. Results and Discussion

Table 1 shows the evolution of the Mohor gazelle and Scimitar-horned oryx populations in RF Guembeul according to published information. The gazelle population grew from 7 to 13 individuals (6 males: 7 females) from 1984 to December 1992. Eighteen deaths were recorded during this eight year period (see [14] for more details on yearly evolution of the population and herd management). From 1992 to 2002 the Mohor gazelle population seemed to grow gradually to 49 [15]. The Senegalese DPN took advantage of the increased population of this gazelle at RF Guembeul to transfer nine of them (2 males: 7 females) to the Réserve de Faune du Ferlo Nord in 2003 (482.000 ha of protected Sahel in Northern Senegal [20,31]). In 2005, only 20 Mohor gazelles were left in RF Guembeul [20] representing the population’s first recorded decrease. We have been unable to find published information on the evolution of the Mohor population in RF Guembeul from 2005 to 2009, when, as we were informed by DPN technicians, only 13–15 gazelles were left in the Reserve (see also [16]). 

**Table 1:** Evolution of the Mohor gazelle and Scimitar-horned oryx populations at RF Guembeul since their respective years of reintroduction (1984 and 1999) according to published information. References are included.

Species	Date	Population size	Reference
*N. d. mhorr*	1984	8	[14]
	1992	13	[14]
	2001	44	[32]
	2002	49	[15]
	2003	(40) 9 individuals to Ferlo	[16,20]
	2005	20	[20]
	2009	15	[16]
	2011	10	Current study
*O. dammah*	1999	8	[18,19]
	2001	14	[19]
	2002	23	[15]
	2003	26	[18,19]
	2003	(18) 8 individuals to Ferlo	[20]
	2004	18	[19]
	2005	18	[20]
	2009	40	[16]
	2011	70	Current study

Available information on the reintroduction of the Scimitar-horned oryx in RF Guembeul tells a different story. It started 15 years later than the Mohor; reinforcement (2:2 individuals) took place after initial reintroduction (3:5); the population has progressively evolved up to now even though eight individuals (2:6) were taken from RF Guembeul to Ferlo Nord in 2003 within the broad DPN initiative for reintroducing Sahel Saharan antelopes there, including the Mohor gazelle.

We found that West and East RF Guembeul had different habitat structures (Table 2). Tree cover, *Opuntia* cover and grass height were significantly different whether sampled in January or in November (no significant interaction). East RF Guembeul is a more open habitat, where trees and *Opuntia* are less abundant (mean tree cover = 38.6%; mean *Opuntia* cover = 0.48%) than in the West (mean tree cover = 71.43%; mean *Opuntia* cover = 17.85%), and the canopy is significantly less developed. Grass height is also lower in the East (mean grass height = 35 cm; in West Guembeul mean grass height = 56.9 cm). The discriminant function correctly classified 100% of plots sampled in East RF Guembeul and 97% in the West. 

We found the same Mohor gazelle population size on both census days. In the East there are 10 individuals distributed as follows: one breeding group formed by one adult male and seven females, and two solitary males. The Scimitar-horned oryx population, on the other hand, was composed of 46 individuals in this part of RF Guembeul; most of them in a large herd (n = 37) with a dominant male, several younger males and many females and their calves. We also detected two family groups of five and three individuals respectively, and one solitary male. In the West, we counted up to 24 individuals, all included in a single herd. This means that the Scimitar-horned oryx population at RF Guembeul had grown to 70 individuals by November 2011.

**Table 2:** Results of the General Discriminant Analysis performed to test for differences in habitat structure between East and West RF Guembeul. The interaction between sampling dates (January *vs.* November) and the three significant variables are also shown.

Variable	F	Effect df	Error df	*p*
Species richness	0.072	1	18	0.792
Bare Ground	1.612	1	18	0.220
*Opuntia* cover	21.289	1	18	**<0.001**
Tree cover	12.849	1	18	**0.002**
Grass cover	2.207	1	18	0.155
Tree height	0.021	1	18	0.886
Grass height	18.981	1	18	**<0.001**
Minimum browsing height	3.316	1	18	0.085
Sampling date (January *vs.* November)	0.904	1	18	0.354
Sampling date* *Opuntia* cover	0.026	1	20	0.873
Sampling date* Tree cover	0.002	1	20	0.969
Sampling date* Grass height	0.008	1	20	0.930

The Mohor gazelle population that was reintroduced in RF Guembeul has experienced a decline in recent years after a post-release increase up to 2002–2003. This gazelle is a desert/semi-desert species [8]. The presence and density of trees appears to condition its distribution throughout its range. Numerous observations have been made in the Atlantic-Sahara desert, mostly in open habitats [33,34,35]. In Niger, its strong preference has been documented for the major “wadis” and their flood plains, secondarily for the steppes in zones of water movement and the dunes invading the “wadi” beds [28]. In Termit Massif it occupies rocky, open areas and sandy fields of barkhan with green pastures and other annual plants [36]. As predicted from its ecological preferences, we found Mohor gazelles currently absent in those zones of RF Guembeul with denser canopy, and where grass is taller, occupying preferably areas characterized by more open, simpler habitats with shorter grass height. This result led us to suggest the possibility that there have been changes in habitat structure during this time in Guembeul, which have reduced the amount of suitable habitat for this gazelle in the Reserve. 

**Figure 2:**
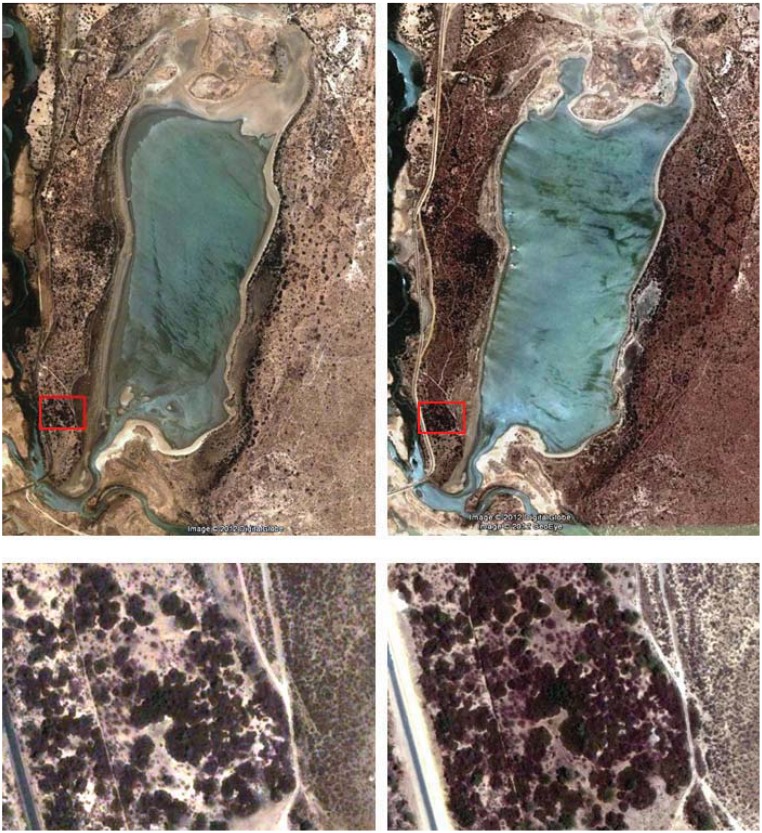
Photos showing changes occurred in vegetation cover in RF Guembeul: left side pictures taken in 13 March 2003; right side pictures taken in 5 April 2011. General view is shown above; details of plots in red, below. Photos have been taken from Google©; accessed on 28 May 2012.

A few months after creation, RF Guembeul was completely fenced off to protect the area from livestock grazing. At that time, it was a rather impoverished sandy habitat due mainly to overgrazing. However, fencing promoted recovery of vegetation, and four years later important changes in its landscape were reported [14]: In 1984 “*Acacia* spp shoots about 25–30 cm high were scattered around the Reserve, with very few mature trees”. In 1988 “the acacias and other trees had grown to a height of 3–5 m and were distributed fairly evenly some 6–10 m apart” and “a small area to the south-west is thickly covered by *Opuntia tuna*”. This has also changed with time, and this cactus is much more widely extended, especially in the West where it appeared in all plots sampled (Table 3). Unfortunately there is no quantified data available on habitat structure in the RF Guembeul in the past that would provide an accurate image of the hypothesized habitat changes over the past years, and which presumably could explain Mohor gazelle decline parallel to an increase in vegetation cover. However, by using historical as well as current Google© images of the Reserve (Figure 2), we can observe changes in the extent of the canopy roughly by comparing pictures taken in 2003 and pictures taken in 2011, and this change is much more pronounced in West Guembeul, very likely associated with the expansion of *Opuntia* in this part of the Reserve. Remote sensing techniques could probably be used in a future follow up study to underpin our current results. 

**Table 3:** Main tree and thorn-bush species found within the sampled plots. Plots are coded as in Figure 1 (1E–7E, eastern part of RF Guembeul; 1W–7W western part). Abbreviations: OT, *Opuntia tuna;* Asp, *Acacia* sp; BA, *Balanites aegyptiaca;* GT, *Grewia tenax*; CP, *Cocculus pendulus*; AD, *Adansonia digitalis*; PJ, *Prosopis juliflora;* SP, *Salvadora persica*; EB, *Euphorbia balsamifera*; CA, *Commiphora africana*; BS, *Boscia senegalensis*; TS, *Tamarix senegalensis*.

Plot	OT	Asp	BA	GT	CP	AD	PJ	SP	EB	CA	BS	TS
1E	X						X		X			
2E		X	X	X						X		
3E		X		X		X	X	X	X	X		
4E												X
5E		X				X	X	X		X	X	
6E		X		X			X	X				X
7E	X	X					X	X				
1W	X	X					X		X			
2W	X	X	X				X					
3W	X	X	X		X			X		X		
4W	X	X	X						X	X		
5W	X	X	X				X	X		X		
6W	X	X		X			X	X				X
7W	X	X					X			X		

It is of interest to analyse the likely causes explaining differences in outcomes of the Scimitar-horned oryx reintroduction relative to the outcomes of the Mohor gazelle reintroduction. Contrary to the latter species, the reintroduced Scimitar-horned oryx population has been increasing progressively in RF Guembeul since 1999. This arid grassland species occupies the same ecological zones as the Mohor gazelle, and the ecology of both species was very similar when sharing their historical range [8]. However, changes observed in habitat structure in our study site do not seem to have had effects on the Scimitar-horned oryx population. One likely explanation is competition. The fact that the Mohor gazelle population decline occurs roughly in parallel to the Scimitar-horned oryx population increase seems to support this explanation. Theory suggests that species that share ecological features may compete. Competing species are expected to develop ways to limit competitive interactions, e.g., niche shift [37]. The Scimitar-horned oryx being much bigger (150–165 kg, [38]), might have taken advantage displacing the smaller Mohor gazelle from their most preferred sites in RF Guembeul, the latter species responding to the presence of the competitor by restricting its niche to a smaller subset (*i.e.*, West Guembeul) of the habitat than it would otherwise do (*i.e.*, the whole Reserve). Alternatively, competing species may reduce interactions by partitioning the available resources (niche partitioning, [39]). Although there does not seem to be any first-hand information on the precise ecology of the Scimitar-horned oryx in its entire ecological zone, but rather that it has been described by extrapolation of the Sahelian information combined with other sparse data [8], it seems that in Niger this species assures shade (an essential element of its habitat during the hot months) by accessing thickly wooded sites. Here *Commiphora africana*, various acacias, and several other Sahelian trees form fairly dense woods in its preferred zones of occupation [8]. Moreover, contrary to Mohor gazelle, during the dry season the Scimitar-horned oryx can feed on succulent plants as a water provider [8]. It is plausible to argue that under a niche partitioning scenario to avoid competition, Mohor gazelle and Scimitar-horned oryx could coexist at Guembeul in the past, populations of both species growing in accordance with their respective ecological demands. However, habitat changes reported in this study, very likely has facilitated the Scimitar-horned population increase and impeded to some extent that of the Mohor gazelle. If this were the case, changes in habitat structure occurred in RF Guembeul in the last 9–10 years could be still claimed as the proxy of the Mohor population decline.

There are other, non-mutually exclusive possibilities for explaining Mohor gazelle decline in Guembeul that might exist: inbreeding, diseases, stochastic effects, and drought together with the inability to move to follow sporadic rainfall in the fenced area of Guembeul, predation. Very likely, there is not one sole factor affecting its decline, and all these alternative hypotheses should be tested to establish the precise causes of its population decrease. High standard data sets to test them all are not available yet. However, this study is only intended to be the very first step of a post-release monitoring agenda that should be developed to gather those key parameters enabling us to evaluate the outcome of the Mohor reintroduction programme which took place in Senegal in 1984. 

## 4. Conclusions

Among the number of complex dilemmas faced by conservationists and managers of reintroduction programmes, the definition of “success” is of major importance. There is no agreement on what constitutes successful reintroduction, although several definitions have been discussed [40], including breeding by the first wild-born generation [41], a three-year breeding population with recruitment exceeding adult death rate [42], and an unsupported wild population of at least 500 individuals [43] among others. Following IUCN Guidelines [44], a reintroduction project attempts to re-establish species within their historical ranges through the release of wild or captive-bred individuals following extirpation or extinction in the wild. As the re-establishment of the species is its final goal, for the purpose of this study we consider reintroduction to be successful if it achieves establishment of a self-sustaining population [45], even if it takes a long time [46]. Accordingly, the reintroduction of the Mohor gazelle in RF Guembeul cannot yet be considered successful, as its population size is presently declining. Our current study is a post-release initiative to investigate the likely reasons for that decline in the interest of bringing the reintroduction programme initiated in 1984 to a successful end. Although reinforcement of the population at RF Guembeul has been recommended elsewhere [8], the likely causes of failure must be identified so that, as strongly recommended by the IUCN [44], those causes may be removed first. In the light of our results, it seems that a key to the long-term survival of the Mohor gazelle in the Reserve is maintenance of a minimum area of suitable habitat in it, avoiding distinct changes in the extent of vegetation cover. Clearing vegetation by periodically cutting down some trees might be an appropriate management policy in Guembeul. These actions could represent a community-based initiative that would significantly increase the effectiveness of biodiversity conservation in Guembeul as for people living around the Reserve its value would be enhanced for them as a job provider. For local authorities this could also be a way to promote sustainable livelihoods for resident populations as well as improve local involvement in management of protected areas. 

Reintroduction should not be undertaken purely as a management practice, but be designated to meet research objectives as well [3], testing hypotheses associated with reintroduction [47,48,49,50]. While reintroduction design usually gives much more attention to short-term factors that may affect survival of the released animals and their descendants (short-term achievement), the key factors for assessing its success may be those that affect the long-term evolution of the population (e.g., the observed changes in habitat structures in RF Guembeul). Most reintroduction projects take place in protected areas, many of them after the area has been fenced in to protect it from livestock grazing. By doing this, wild vegetation is recovered, assuring food resources for animals. However, sometimes protection also has detrimental effects, as is the case of the excessive development of canopy in our Senegalese study area.
